# The Effect of Seat Back Inclination on Spinal Alignment in Automotive Seating Postures

**DOI:** 10.3389/fbioe.2021.684043

**Published:** 2021-08-02

**Authors:** Fusako Sato, Yusuke Miyazaki, Shigehiro Morikawa, Antonio Ferreiro Perez, Sylvia Schick, Karin Brolin, Mats Svensson

**Affiliations:** ^1^Safety Research Division, Japan Automobile Research Institute, Tsukuba, Japan; ^2^Department of Mechanics and Maritime Sciences, Chalmers University of Technology, Gothenburg, Sweden; ^3^Department of Systems and Control Engineering, Tokyo Institute of Technology, Tokyo, Japan; ^4^Shiga University of Medical Science, Otsu, Japan; ^5^Fundación de Investigación HM Hospitales, Madrid, Spain; ^6^Department of Forensic Epidemiology, Institute of Legal Medicine, Ludwig Maximilian University of Munich, Munich, Germany; ^7^Lightness by Design Aktiebolag (AB), Stockholm, Sweden

**Keywords:** automotive seating posture, MRI, multi-dimensional scaling, seat back inclination, spinal alignment, spinal injury, spinal segmental angle

## Abstract

Experimental studies have demonstrated a relationship between spinal injury severity and vertebral kinematics, influenced by the initial spinal alignment of automotive occupants. Spinal alignment has been considered one of the possible causes of gender differences in the risk of sustaining spinal injuries. To predict vertebral kinematics and investigate spinal injury mechanisms, including gender-related mechanisms, under different seat back inclinations, it is needed to investigate the effect of the seat back inclination on initial spinal alignment in automotive seating postures for both men and women. The purpose of this study was to investigate the effect of the seat back inclination on spinal alignments, comparing spinal alignments of automotive seating postures in the 20° and 25° seat back angle and standing and supine postures. The spinal columns of 11 female and 12 male volunteers in automotive seating, standing, and supine postures were scanned in an upright open magnetic resonance imaging system. Patterns of their spinal alignments were analyzed using Multidimensional Scaling presented in a distribution map. Spinal segmental angles (cervical curvature, T1 slope, total thoracic kyphosis, upper thoracic kyphosis, lower thoracic kyphosis, lumbar lordosis, and sacral slope) were also measured using the imaging data. In the maximum individual variances in spinal alignment, a relationship between the cervical and thoracic spinal alignment was found in multidimensional scaling analyses. Subjects with a more lordotic cervical spine had a pronounced kyphotic thoracic spine, whereas subjects with a straighter to kyphotic cervical spine had a less kyphotic thoracic spine. When categorizing spinal alignments into two groups based on the spinal segmental angle of cervical curvature, spinal alignments with a lordotic cervical spine showed significantly greater absolute average values of T1 slope, total thoracic kyphosis, and lower thoracic kyphosis for both the 20° and 25° seat back angles. For automotive seating postures, the gender difference in spinal alignment was almost straight cervical and less-kyphotic thoracic spine for the female subjects and lordotic cervical and more pronounced kyphotic thoracic spine for the male subjects. The most prominent influence of seatback inclination appeared in Total thoracic kyphosis, with increased angles for 25° seat back, 8.0° greater in spinal alignments with a lordotic cervical spine, 3.2° greater in spinal alignments with a kyphotic cervical spine. The difference in total thoracic kyphosis between the two seatback angles and between the seating posture with the 20° seat back angle and the standing posture was greater for spinal alignments with a lordotic cervical spine than for spinal alignments with a kyphotic cervical spine. The female subjects in this study had a tendency toward the kyphotic cervical spine. Some of the differences between average gender-specific spinal alignments may be explained by the findings observed in the differences between spinal alignments with a lordotic and kyphotic cervical spine.

## Introduction

In investigations of spinal injury biomechanics in road traffic accidents, it has been considered that cervical spinal alignment is one potential factor that may influence the severity of the cervical spinal injury. Experimental studies using human cadavers have demonstrated the influence of the initial cervical spinal alignment on the severity of cervical spinal injuries (Maiman et al., 1983, 2002; Yoganandan et al., 1986, 1999; Liu and Dai, 1989; Pintar et al., 1995). Because of load transmission between the head and the torso through the cervical spine, cervical spinal alignment can affect vertebral translational and rotational kinematics during impact. One computational study using a head-neck model found that kyphotic cervical spinal alignment was exposed to larger elongation of the facet joint capsular ligaments than lordotic cervical spinal alignment in rear impact loadings (Stemper et al., 2005). Therefore, the study concluded that a kyphotic cervical spine has a more potentially harmful effect on the risk of sustaining cervical spinal injuries. Indeed, a series of human volunteer rear impact sled tests showed that cervical vertebrae with kyphotic cervical spinal alignment rotated significantly more in extension than cervical vertebrae with lordotic cervical spinal alignment (Ono et al., 1997).

Another series of human volunteer rear impact sled tests have indicated the importance of interaction between the torso and seat back on cervical spinal kinematics (Ono et al., 1999). In computational studies using a whole-body human finite element (FE) model, the initial thoracolumbar spinal alignment influenced vertebral kinematics of the whole spine in rear impact reconstructions (Sato et al., 2010, 2017). Thoracolumbar vertebral kinematics govern the T1 kinematics, which can directly affect cervical spinal kinematics. Therefore, it seems that the initial whole spinal alignments are essential factors for clarifying spinal injury mechanisms.

Epidemiologic studies have shown that women are at a higher risk to sustain cervical spinal injuries, including whiplash-associated disorders (WADs), in traffic accidents compared with men (Kihlberg, 1969; O'Neill et al., 1972; Thomas et al., 1982; Otremski et al., 1989; Maag et al., 1990; Morris and Thomas, 1996; Dolinis, 1997; Temming and Zobel, 1998; Chapline et al., 2000; Richter et al., 2000; Krafft et al., 2003; Jakobsson et al., 2004; Storvik et al., 2009; Carstensen et al., 2012; Forman et al., 2019a). The gender differences in the risk of sustaining cervical spinal injuries are attributed partly to anatomical, biomechanical, and muscular differences between men and women (Stemper et al., 2011; Stemper and Corner, 2016). In the gender-dependent anatomical differences, cervical spinal alignment has been considered one of the possible causes of gender differences in the risk of sustaining cervical spinal injuries (Helliwel et al., 1994; Matsumoto et al., 1998; Stemper et al., 2011; Brolin et al., 2015; Stemper and Corner, 2016; Östh et al., 2017; Sato et al., 2017; John et al., 2018). In an asymptomatic population measured in an upright seated position, cervical lordosis was observed in the majority, and non-lordotic alignment was observed in 36% (Matsumoto et al., 1998) and 38% (Takeshima et al., 2002). Women are more likely to present non-lordosis (straight or kyphosis) than men, while men statistically present more pronounced lordosis (Helliwel et al., 1994; Hardacker et al., 1997; Matsumoto et al., 1998; Been et al., 2017).

Rear impact sled tests using head-neck complexes extracted from cadavers have demonstrated greater intervertebral angular displacements and shear displacements between facet joints for female specimens than for male specimens (Stemper et al., 2003, 2004). Using a FE model of the C5–C6 spinal segment, computational simulations based on the experiments conducted by Stemper et al. (2003) have shown that the straighter C5–C6 spinal segment exhibited greater posterior facet joint compression and anterior longitudinal ligament stretch (John et al., 2018). Consequently, the study concluded that these findings might explain the higher risk of sustaining cervical spinal injuries for women with a straighter cervical spine. However, these studies have been limited to the cervical spine region.

Human volunteer sled tests have also demonstrated greater intervertebral flexion in the upper cervical spine and greater intervertebral extension in the lower cervical spine (Ono et al., 2006; Sato et al., 2014, 2015). The whole spinal alignment, from C2 to the sacrum, was investigated in the same seating posture and the same seat configuration as the human volunteer sled tests conducted by Ono et al. (2006) and Sato et al. (2017). The study has reported straighter spinal alignment, almost straight cervical, and less-kyphotic thoracic spine for female subjects. By changing the spinal alignment of a whole-body human FE model, reconstruction simulations of the human volunteer sled tests have illustrated the female spinal alignment exhibited greater intervertebral flexion in the upper cervical spine and greater intervertebral extension in the lower cervical spine, explaining the influence of the interaction between thoracolumbar spine and seat back on cervical vertebral kinematics. These studies were conducted using a laboratory seat with a 20° seat back angle.

Basically, the seat performance of occupant protection systems installed in cars is evaluated at a seat back angle of 25° (SAE Standard J826, 2015). Furthermore, highly automated vehicles have the potential to allow drivers in a reclined position (Forman et al., 2019b; Gepber et al., 2019). It is important to evaluate vertebral kinematics of a reclined spinal alignment for future crash safety with highly automated vehicles. Human cadaver sled tests have shown the effect of the seat back inclination on cervical vertebral rotations and facet joint shear displacements (Deng et al., 2000; Yang and King, 2003). To predict vertebral kinematics and investigate spinal injury mechanisms, including gender-related mechanisms, under different seat back inclinations, it is needed to investigate the effect of the seat back inclination on initial spinal alignment in automotive seating postures for both men and women.

In the past, whole spinal alignments have been studied through medical imaging data, either in a standing (Hardacker et al., 1997; Janssen et al., 2009; Ames et al., 2013; Park et al., 2013) or supine position (Parenteau et al., 2014). To be relevant for traffic safety research, it is important that spinal alignments are characterized in postures representative for male and female automotive occupants (hereafter referred to as automotive seating postures) (Chabert et al., 1998; Klinich et al., 2004, 2012; Reed and Jones, 2017; Sato et al., 2017, 2019; Izumiyama et al., 2018). Chabert et al. (1998) showed the whole spinal alignment of an automotive seating posture for one male human cadaver. Klinich et al. (2004, 2012) and Reed and Jones (2017) analyzed cervical spinal alignments in one automotive seating posture with a 19° seat back angle for 180 male and female volunteers. Recently, Sato et al. (2017, 2019) investigated representative spinal alignments from C2 to the sacrum and the relationship between the cervical, thoracic and lumbar spinal alignment for male and female volunteers in one automotive seating posture with a 20° seat back angle, as described above. However, as these studies only investigated a single automotive seating posture, it remains to be determined how different seat back inclinations affect initial whole spinal alignment for both male and female automotive occupants.

The purpose of this study was to investigate the effect of seat back inclination on the spinal alignment of automotive seating postures for both men and women. This study targeted a 20° seat back angle, which has been investigated in our previous studies (Sato et al., 2017, 2019), and a 25° seat back angle, which is used in car crash tests (SAE Standard J826, 2015). In addition, spinal alignments in automotive seating postures were compared with supine postures to provide information about spinal alignment in reclined automotive seating postures for highly automated vehicles. Spinal alignment of a standing posture was also compared with obtain fundamental knowledge of spinal alignment based on previous studies in the medical field.

## Materials and Methods

The effect of seat back inclination on spinal alignment was investigated by comparing automotive seating postures in 20° and 25° seat back angles and standing and supine postures.

The spinal columns of volunteers in the seating, standing, and supine postures were scanned in an upright open magnetic resonance imaging (MRI) system. The MRI dataset of the automotive seating posture in the 20° seat back angle for eight female and seven male subjects, as listed in Groups 1 and 2 in Table 1, were obtained from our previous study (Sato et al., 2017). The automotive seating posture with the 20° seat back angle was set to the same seating posture and the same seat configuration as in a series of volunteer rear impact sled tests (Ono et al., 2006). The volunteers in Groups 1 and 2 were also subjected to MRI scans in standing and supine postures. Additional MRI datasets were acquired for three female and five male subjects, Group 3 in Table 2, in the automotive seating postures with the 20° and 25° seat back angles and standing and supine postures. The seat back angle of 25° was applied based on the crash test dummy positioning (SAE Standard J826, 2015).

**Table 1:** Test groups and subjects.

**Group ID**	**Seatback angle (deg)**	**Posture**	**Sex**	**No. of subjects**	**Height** **(cm)**	**Weight** **(kg)**
1 (Japanese)	20	Supine	Female	5	159.9 (5.3)	47.8 (6.1)
			Male	3	171.4 (0.7)	64.5 (4.9)
2 (European)	20	Standing, Supine	Female	3	162.3 (4.4)	58.3 (2.3)
			Male	4	175.2 (0.5)	77.7 (4.5)
3 (European)	20, 25	Standing, Supine	Female	3	162.7 (2.1)	58.3 (3.2)
			Male	5	175.8 (1.6)	78.0 (3.5)

*Average and SD in brackets*.

**Table 2:** Angular measurements of spinal segments.

**Angular measurements**	**Description**
Cervical curvature (CC)	Angle between C2 and C7 (Harrison et al., 2000)
T1 slope (TS)	Angle of T1 from the horizontal line (Rocabado, 1983; Armijo-Olivo et al., 2006; Park et al., 2015)
Total thoracic kyphosis (TTK)	Angle between T1 and T12 (Rocabado, 1983; Armijo-Olivo et al., 2006; Park et al., 2015)
Upper thoracic kyphosis (UTK)	Angle between T1 and T4 (Rocabado, 1983; Armijo-Olivo et al., 2006; Park et al., 2015)
Lower thoracic kyphosis (LTK)	Angle between T4 and T12 (Rocabado, 1983; Armijo-Olivo et al., 2006; Park et al., 2015)
Lumbar lordosis (LL)	Angle between L1 and sacrum (Rocabado, 1983; Armijo-Olivo et al., 2006; Park et al., 2015)
Sacral slope (SS)	Angle of sacrum from the horizontal line (Rocabado, 1983; Armijo-Olivo et al., 2006; Park et al., 2015)

Spinal alignments were extracted from the MRI dataset. To visually describe the overall trend of spinal alignment, representative patterns of spinal alignment in each posture, including average gender-specific spinal alignments, were analyzed with multidimensional scaling (MDS), presenting a distribution map (Cox and Cox, 2000; Mochimaru and Kouchi, 2000; Borg and Groenen, 2005; Miyazaki et al., 2005), as described in Section Spinal Alignment Patterns and in detail in our previous study (Sato et al., 2017). The variation in spinal alignment due to individual differences in each posture was studied through MDS analyses, and the average gender-specific spinal alignments were compared between postures.

Spinal segmental angles were measured on the MRI dataset in order to analyse the spinal alignment similar to a commonly used method in previous publications (Rocabado, 1983; Harrison et al., 2000; Berthonnaud et al., 2005; Roussouly et al., 2005; Armijo-Olivo et al., 2006; Mac-Thiong et al., 2007; Park et al., 2015), as described in section Spinal Segmental Angles. To look at the overall trend in spinal alignment from the perspective of the spinal segmental angles, correlations between the spinal segmental angles were analyzed. Thereafter, spinal segmental angles were compared between postures.

All procedures have been approved by the Ethical Committee of Shiga University of Medical Science in Japan, Hospital Universitario HM Montepríncipe (Fundación de Investigación HM Hospitales) in Spain, Japan Automobile Research Institute, and Tokyo Institute of Technology in Japan.

### Human Subjects

Subjects comprised a total of 11 female, and 12 male volunteers divided into three groups, as listed in Table 1. The age of the subjects ranged from 21 to 38 years averaging at 27 years. None of the subjects had any known history of spinal injury. The target height and weight [average ± SD (SD)] for selecting the Japanese subjects were based on the average Japanese female and male body sizes for 20–40 year-olds; 159 ± 5 cm and 51 ± 6 kg for women and 172 ± 6 cm and 67 ± 9 kg for men (Ministry of Education, 2013). For European subjects, the target height and weight were defined based on the 50th percentile female and male body sizes, as reported in the University of Michigan Transportation Research Institute study (Schneider et al., 1983); 161.8 cm and 62.3 kg for women and 175.3 cm and 77.3 kg for men.

### MRI Acquisition

A non-metallic seat, designed to correspond to the seat of the volunteer sled tests (Ono et al., 2006) was installed in an upright open MRI system, Signa SP2 (GE Healthcare Inc., Madison, WI) at Shiga University of Medical Science and in a Fonar Upright Multi-Position MRI system (Fonar Inc., Melville, NY) at Hospital Universitario HM Montepríncipe. The seat consisted of two flat plates with a 20° or 25° seat back angle from the vertical plane and a 10° seat pan angle from the horizontal plane. As per the procedure in the sled tests (Ono et al., 2006), subjects were instructed to sit on the seat deeply, face forward in a relaxed manner, keeping physical contact from the pelvic level up to the shoulder blades against the seat back. The head was held such that the Frankfort plane angle was ~10° upward from the horizontal plane. The femur lines, defined from the great trochanter to the knee joint center of rotation, were tilted at 25° upward from the horizontal plane. Similarly, subjects were instructed to stand straight and face forward in a relaxed manner for the standing posture, keeping the head with the Frankfort plane angle of ~10° upward from the horizontal plane. For the supine posture, subjects were laid straight on their back on a flat horizontal table. The main acquisitions were carried out with a T1-weighted 3D gradient echo sequence in the sagittal plane. Due to the limitation of the field of view, the full spinal column was scanned in three or four serial images with enough overlap to cut off geometric warping of images at the edge of the field. The volunteer's position in the MRI system was adjusted to fit the field of view for each scan. All MRI scans were conducted at Shiga University of Medical Science for the Japanese subjects and Hospital Universitario HM Montepríncipe for the European subjects.

### Spinal Alignment Patterns

**Figure 1:**
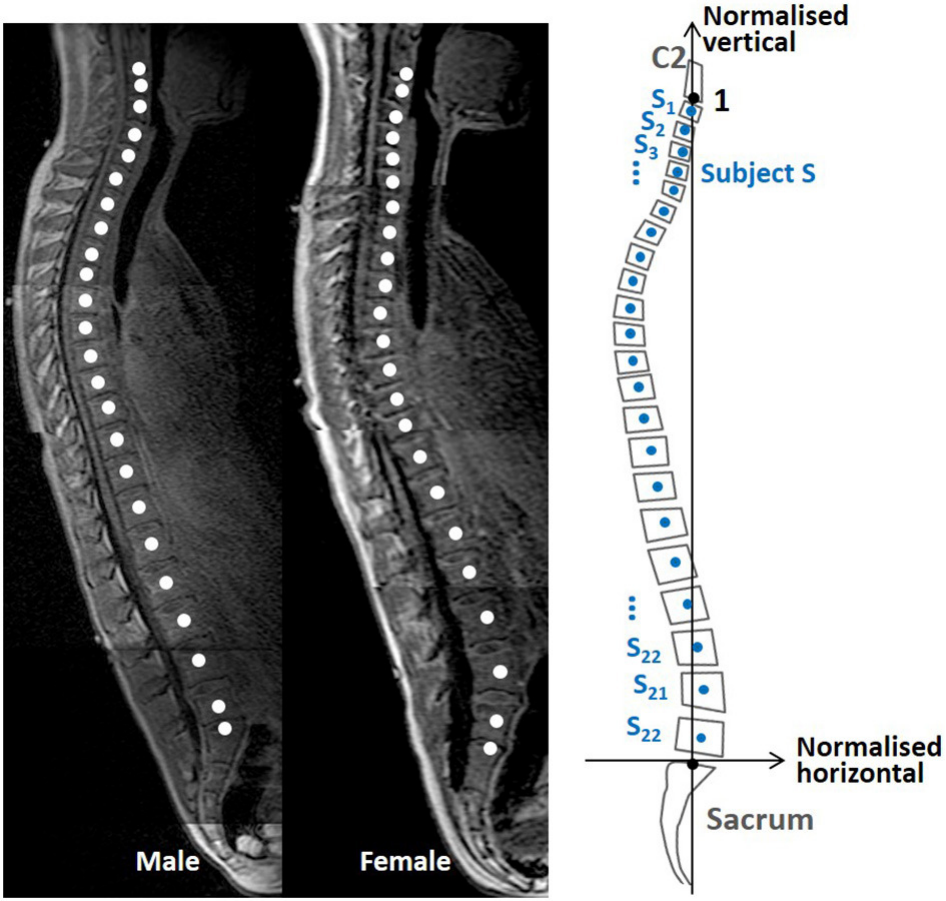
Magnetic resonance imaging data spinal alignment and transfer to a normalized coordinate system.

Spinal alignments in this study were presented with the geometrical centers of the vertebral bodies in midsagittal images, as shown in Figure 1. For C2 and the sacrum, the midpoint of the inferior and superior surface of the vertebral body was used, respectively. The coordinates of these points, used to define spinal alignments, were extracted with the medical imaging software OsiriX (Pixmeo, Geneva, Switzerland). After that, spinal alignments were normalized by the C2-sacrum length and rotated around the sacrum, defined as the origin to move C2 to 1 on the normalized vertical axis.

Spinal alignment patterns were investigated with MDS (Cox and Cox, 2000; Mochimaru and Kouchi, 2000; Borg and Groenen, 2005; Miyazaki et al., 2005). MDS is a statistical method for high-dimensional data to create a distribution map, visualizing similarities between investigated objects by relative positions in reduced data dimensions, generally two or three dimensions less (Cox and Cox, 2000).

A distance matrix *D* in Equation 1, applied as the input data for an MDS analysis, comprised all possible inter-individual distances between two subjects. The inter-individual distance between subjects *S* and *T, e*_*st*_ in Equation (2) was represented as the sum of squared Euclidean pairwise distances between corresponding vertebral points *s*_*i*_ and *t*_*i*_ in the normalized coordinate system.

(1) $$\begin{array}{l} D=\left(\begin{array}{ccc} e_{11} & \cdots & e_{1n}\\ \vdots & \ddots & \vdots \\ e_{n1} & \cdots & e_{nn} \end{array}\right) \end{array}$$

(2) $$\begin{array}{l} e_{st}=\overset{22}{\underset{i=1}{\sum }}{\left(s_{i}-t_{i}\right)}^{2} \end{array}$$

where *n* means the *nth* subject, *i* means the *ith* vertebra from C3 to L5. *s*_*i*_ and *t*_*i*_ mean subject *S* or *T*'s *ith* vertebral point containing normalized horizontal and vertical coordinates, a total of 22 points for each subject. By conducting MDS on the distance matrix *D*, a two-dimensional distribution map of the spinal alignments was obtained, identifying the two MDS dimensions with the largest inter-subject variance in spinal alignment.

In the distribution map, four spinal alignments were estimated as representative spinal alignments for each posture at the intersections of the 50% probability ellipse and the axes of the two MDS dimensions to describe underlying spinal alignment patterns indicated by each MDS dimension. Those spinal alignments were calculated by the weighted average of spinal alignments to minimize the difference between the MDS score of each estimated spinal alignment and the intersection. Similarly, average gender-specific spinal alignments were estimated at the average points for female and male subjects.

### Spinal Segmental Angles

**Figure 2:**
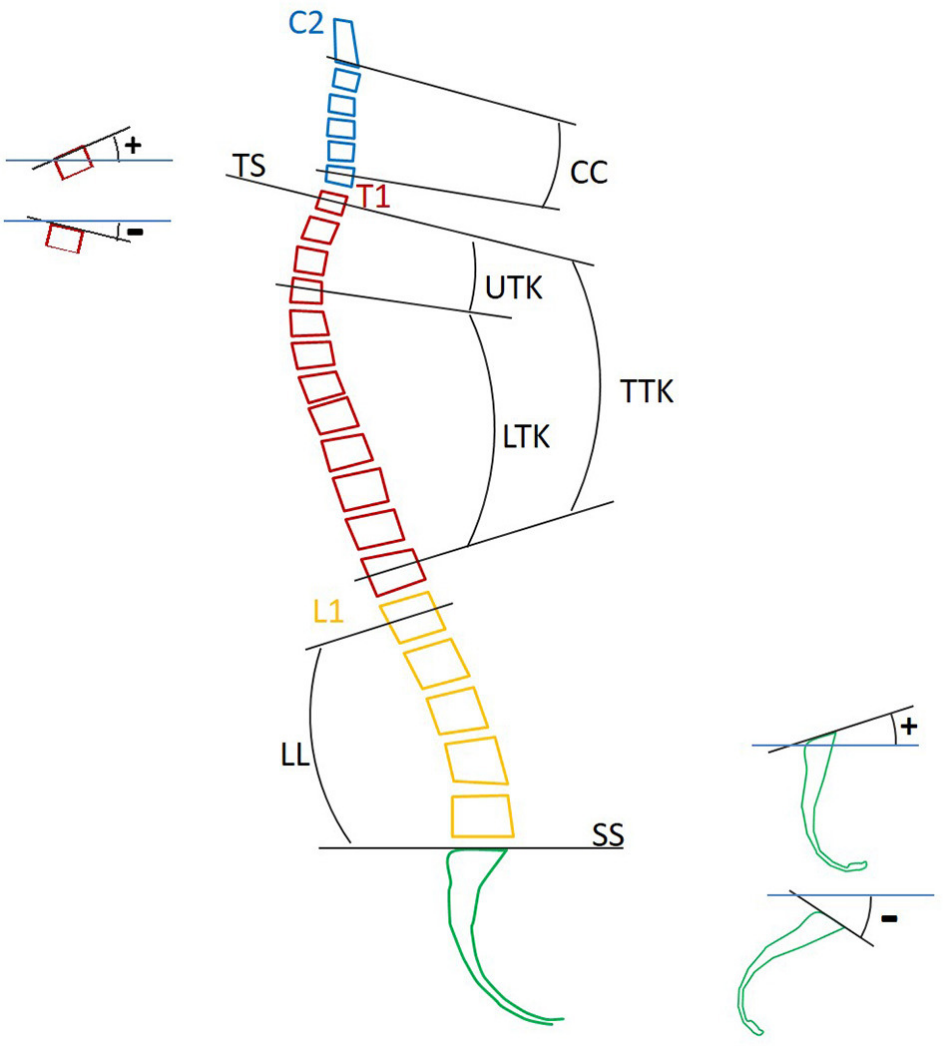
Definitions of the angular measurements for the spinal segments.

In accordance with previous investigations on spinal segmental angles (Rocabado, 1983; Harrison et al., 2000; Berthonnaud et al., 2005; Roussouly et al., 2005; Armijo-Olivo et al., 2006; Mac-Thiong et al., 2007; Park et al., 2015), the spinal segmental angles illustrated in Figure 2 and Table 2 were measured based on the vertebral angles in midsagittal images of the MRI data using the medical imaging software OsiriX (Pixmeo, Geneva, Switzerland). The spinal segmental angles measured in this study are cervical curvature (CC), T1 slope (TS), total thoracic kyphosis (TTK), upper thoracic kyphosis (UTK), lower thoracic kyphosis (LTK), lumbar lordosis (LL), and sacral slope (SS). In this study, each vertebral angle was defined as the angle of the median plane between the superior and inferior surface of the vertebral body on the midsagittal plane. The angle of the inferior and superior surfaces was used for C2 and the sacrum, respectively. The positive angle indicates a lordotic curvature or upward angle from the horizontal plane, while the negative angle indicates a kyphotic curvature or downward angle from the horizontal plane. For each segmental angle, the average and SD were obtained.

## Results

### Spinal Alignment Patterns

#### Automotive Seating Postures in the 20° and 25° Seat Back Angles

**Figure 3:**
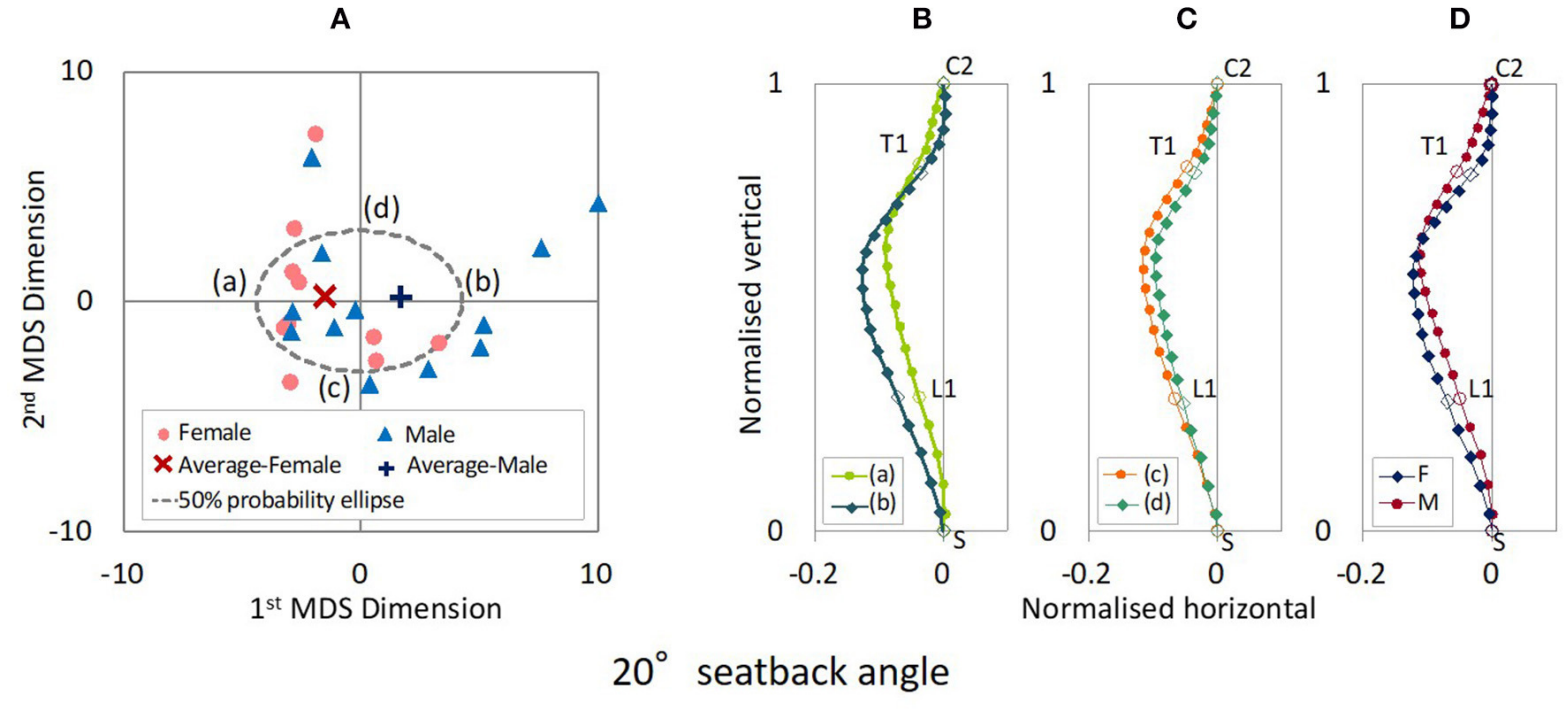
**(A)** MDS distribution map of spinal alignments for the 20° seatback angle. **(B)** Spinal alignments estimated at the intersections of the 50% probability ellipse with the 1st MDS dimension (a,b) and **(C)** the 2nd MDS dimension (c,d), and **(D)** the female and the male average points (F and M).

**Figure 4:**
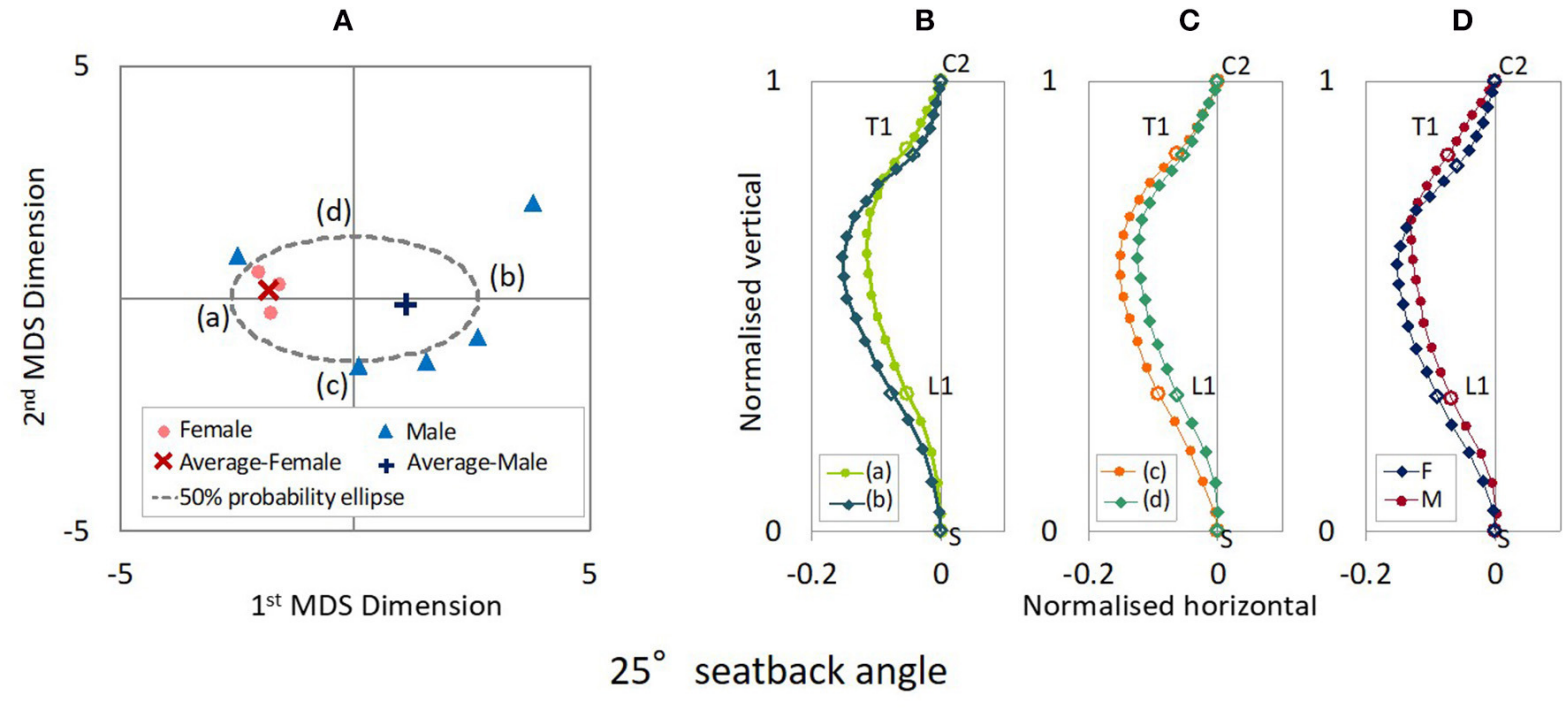
**(A)** MDS distribution map of spinal alignments for the 25° seatback angle. **(B)** Spinal alignments estimated at the intersections of the 50% probability ellipse with the 1st MDS dimension (a,b) and **(C)** the 2nd MDS dimension (c,d), and **(D)** the female and the male average points (F and M).

The distribution maps of spinal alignments in the 20° and 25° seat back angles are illustrated in Figures 3A, 4A. For the 20° seat back angle, the contribution ratio is 62.5% for the first MDS dimension, 31.0% for the second MDS dimension, 4.0% for the third MDS dimension, and 1.9% for the fourth MDS dimension. Limiting the distribution map of spinal alignments to the first two MDS dimensions captured 93.5% of the total inter-subject variance. For the 25° seat back angle, the first to fourth MDS dimensions explained 72.0, 18.2, 8.5, and 1.1% of the total inter-subject variance, respectively. The two-dimensional distribution map consisting of the first two MDS dimensions captured 90.2% of the total inter-subject variance.

Figures 3B,C shows the spinal alignments estimated at the intersection of the 50% probability ellipse with the axes of the first and second MDS dimensions for the 20° seat back angle. The first MDS dimension explains the maximum variance of spinal alignment. Along the first MDS dimension, spinal alignment varies between an almost straight cervical and less kyphotic thoracic spine to a lordotic cervical and more pronounced kyphotic thoracic spine, comparing the spinal alignment (a) and (b) in Figure 3B. The second maximum variance of spinal alignment along the second MDS dimension illustrated that spinal alignment varies the thoracic spine between a rearward to a forward position with similar cervical spinal alignment, comparing the spinal alignment (c) and (d) in Figure 3C. The estimated average spinal alignment for each gender is shown in Figure 3D. On the distribution map, the average MDS point was located on the left side against the origin for female subjects and the right side for male subjects along the first MDS dimension, while the average MDS score of the second MDS dimension was close to zero for both genders. Hence, the estimated average gender-specific spinal alignments illustrated the variation indicated along the first MDS dimension, an almost straight cervical and less-kyphotic thoracic spine for the female subjects, and a lordotic cervical and more pronounced kyphotic thoracic spine for the male subjects. Similar trends in spinal alignment patterns were observed in the variation of spinal alignment for the 25° seat back angle as for the 20° seat back angle, as shown in Figure 4.

**Figure 5:**
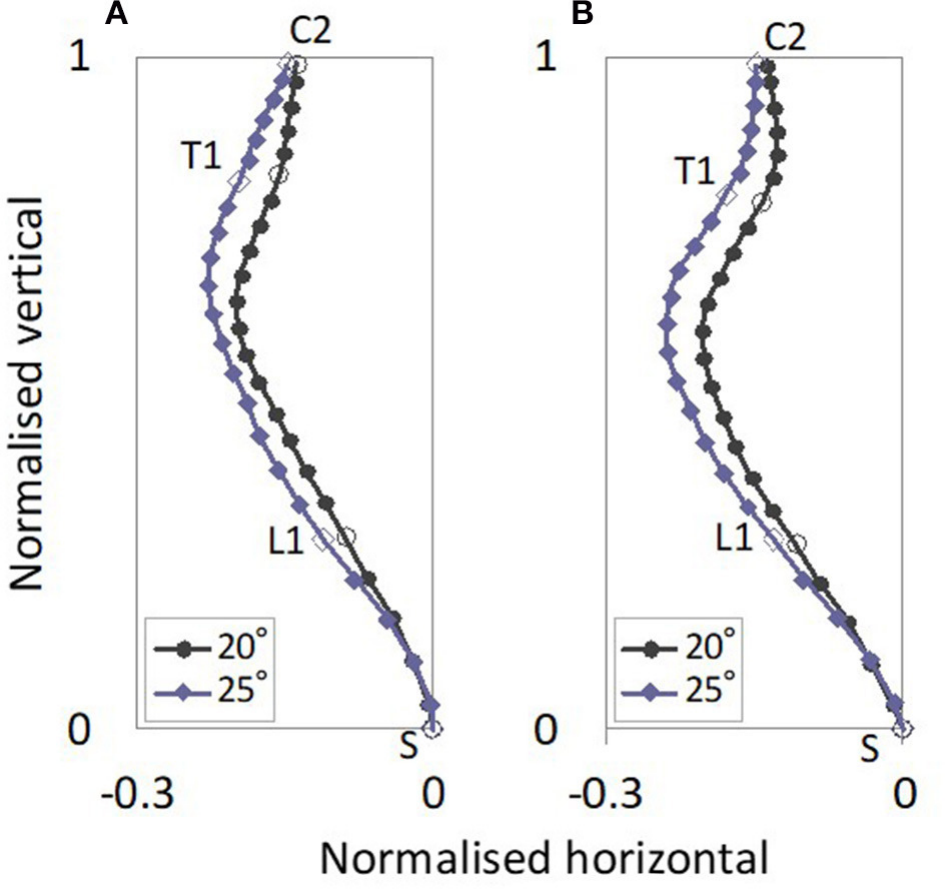
Average gender-specific spinal alignments with 20° and 25° seat back angles. The average gender-specific spinal alignments were rotated back to the original position by average rotational angles for the female and male subjects in each posture, obtained in the normalization of the spinal alignment. **(A)** Female, **(B)** Male.

The estimated average gender-specific spinal alignments are shown in Figure 5. For both the female and male subjects, the spinal alignments in the 25° seat back angle were located rearward of the spinal alignments in the 20° seat back angle from L2 to C2, and came close at C2, exhibiting a similar spinal alignment pattern to that with the 20° seat back angle.

#### Standing and Supine Postures

**Figure 6:**
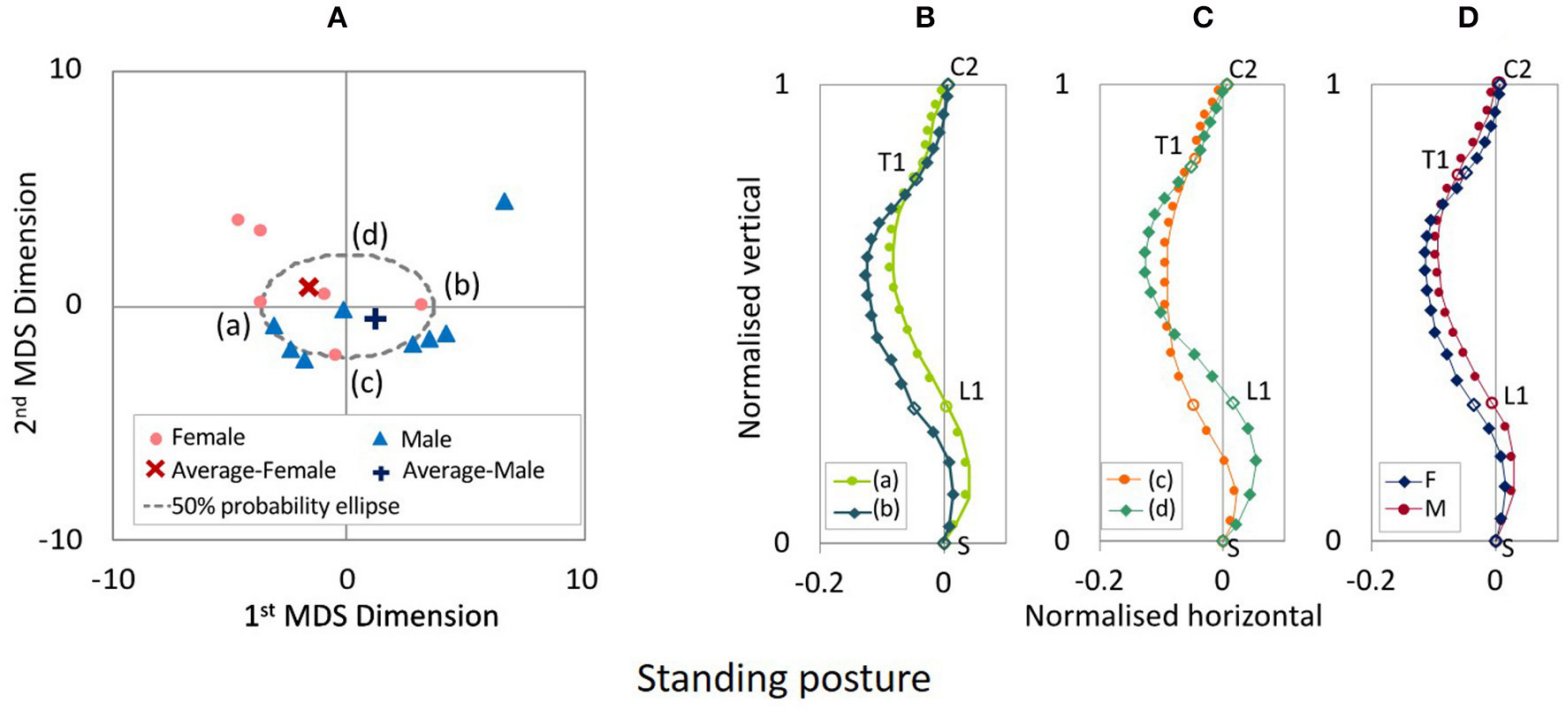
**(A)** MDS distribution map of spinal alignments for the standing posture. **(B)** Spinal alignments estimated at the intersections of the 50% probability ellipse with the 1st MDS dimension (a,b) and **(C)** the 2nd MDS dimension (c,d), and **(D)** the female and the male average points (F and M).

The distribution map of spinal alignments in the standing posture is shown in Figure 6A. The first to fourth MDS dimensions explained 61.7, 22.2, 14.7, and 1.1% of total inter-subject variance, respectively. The two-dimensional distribution map consisting of the first two MDS dimensions captured 83.9% of the total inter-subject variance.

The estimated spinal alignments at the intersection of the 50% probability ellipse with the axes of the first and second MDS dimensions for the standing posture are illustrated in Figures 6B,C. To understand the maximum variance of spinal alignment illustrated by the first MDS dimension, the estimated spinal alignment (a) and (b) in Figure 6B were compared. Along the first MDS dimension, spinal alignment varies between the combination from a less kyphotic thoracic and lordotic lumbar spine with a slightly kyphotic cervical spine to a more pronounced kyphotic thoracic and lordotic lumbar spine with a lordotic cervical spine. The second maximum variance of spinal alignment along the second MDS dimension illustrated that thoracolumbar spinal alignments vary between straighter to more pronounced S-shape spine with similar cervical spinal alignment, comparing the spinal alignment (c) and (d) in Figure 6C. On the distribution map, the average MDS point was located on the left side against the origin for female subjects and the right side for male subjects, along the first MDS dimension. Hence, the estimated average gender-specific spinal alignments were in line with the trend observed along the first MDS dimension, as shown in Figure 6D.

**Figure 7:**
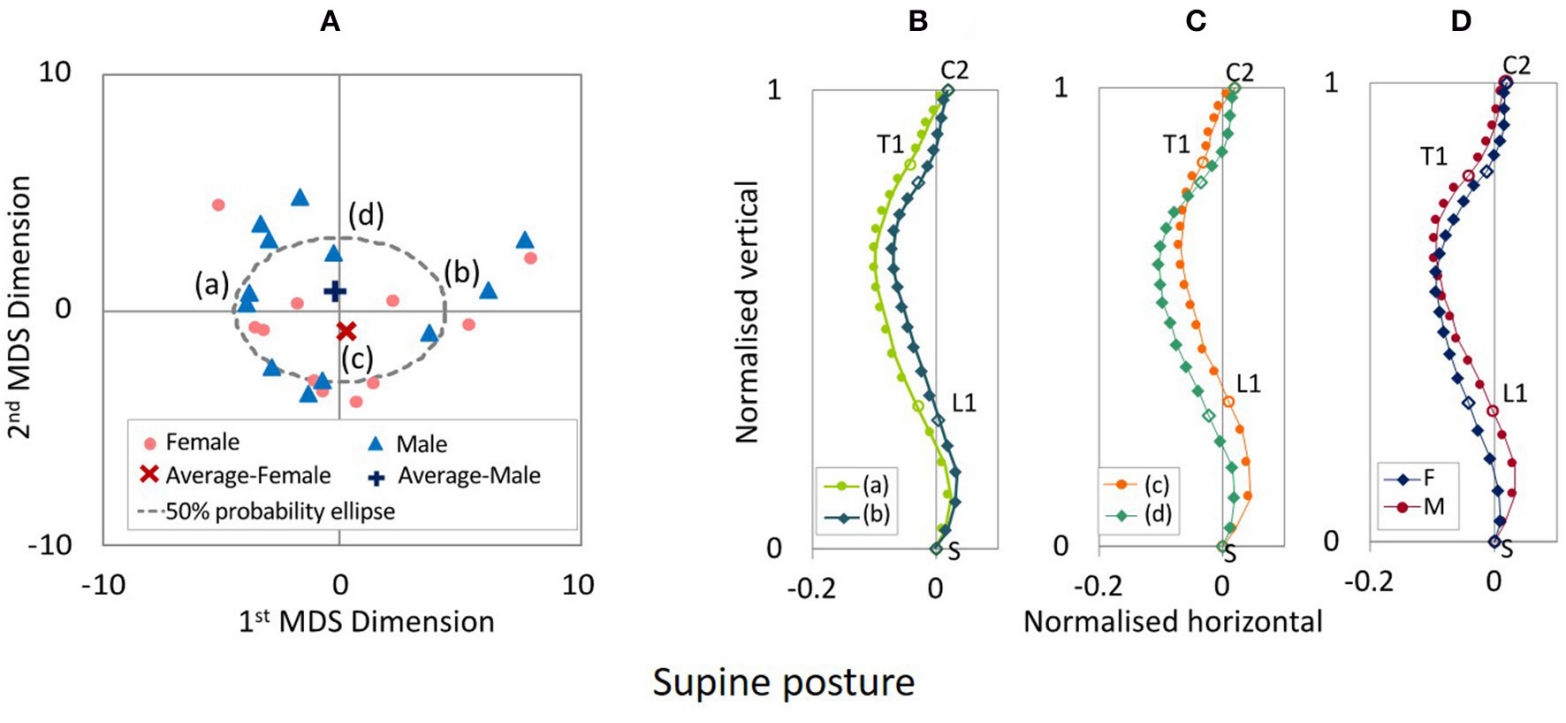
**(A)** MDS distribution map of spinal alignments for the supine posture. **(B)** Spinal alignments estimated at the intersections of the 50% probability ellipse with the 1st MDS dimension (a,b) and **(C)** the 2nd MDS dimension (c,d), and **(D)** the female and the male average points (F and M).

The distribution map of spinal alignments in the supine posture is shown in Figure 7A. The first to fourth MDS dimensions explained 53.4, 25.5, 19.9, and 0.9% of total inter-subject variance, respectively. The two-dimensional distribution map consisting of the first two MDS dimensions captured 78.9% of the total inter-subject variance.

The estimated spinal alignments at the intersection of the 50% probability ellipse with the axes of the first and second MDS dimensions for the supine posture are illustrated in Figures 7B,C. Comparing the spinal alignment (a) and (b) in Figure 7B, the maximum variance of spinal alignment illustrated along the first MDS dimension that spinal alignment varies between a straighter cervicothoracic spine to a more pronounced kyphotic thoracic spine. Along the second MDS dimension, the second maximum variance of spinal alignment illustrated that spinal alignment varies between straighter to more pronounced S-shape thoracolumbar spine with, comparing the spinal alignment (c) and (d) in Figure 7C. The estimated average spinal alignment for each gender is shown in Figure 7D. The average MDS point on the distribution map shown in Figure 7a was located on the lower side against the origin for female subjects and the upper side for male subjects, along the second MDS dimension. Consequently, the estimated average gender-specific spinal alignments were consistent with the trend observed along the second MDS dimension, as shown in Figure 7D.

**Figure 8:**
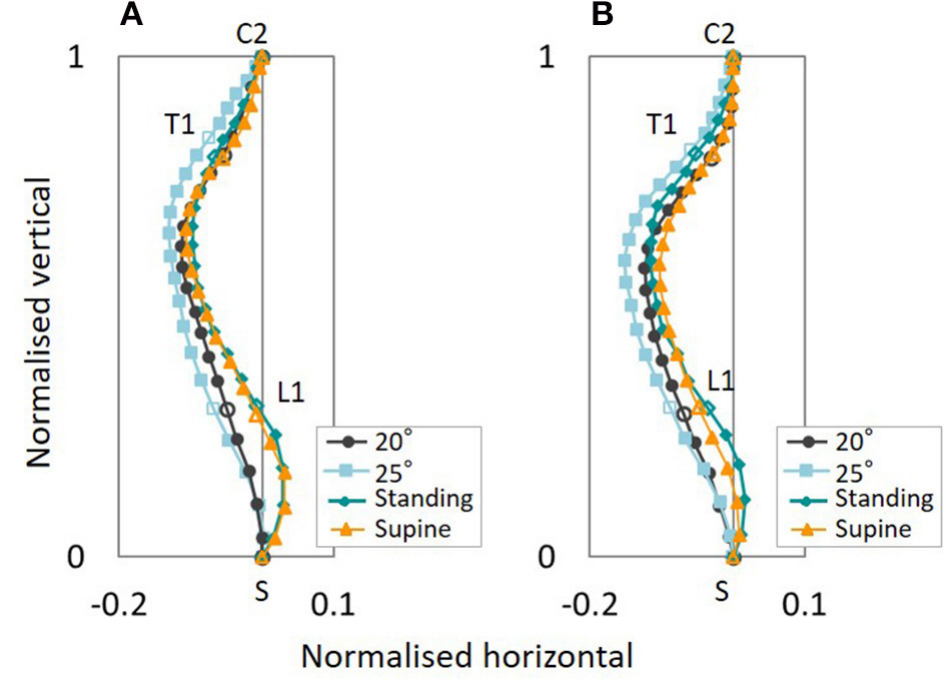
Average gender-specific spinal alignments for the standing and supine postures, comparing the automotive seating posture with the 20° and 25° seat back angle. **(A)** Female, **(B)** Male.

The estimated average gender-specific spinal alignments of the standing and supine postures, comparing spinal alignments of the automotive seating posture in the 20° and 25° seat back angle, are shown in Figure 8. For both the female and male subjects, the cervical and upper thoracic spine exhibited similar spinal alignment in the four postures, whereas the lumbar spine showed more pronounced lordosis for the standing and supine postures than for the seating postures.

### Spinal Segmental Angles

#### Correlations Between Spinal Segmental Angles

**Figure 9:**
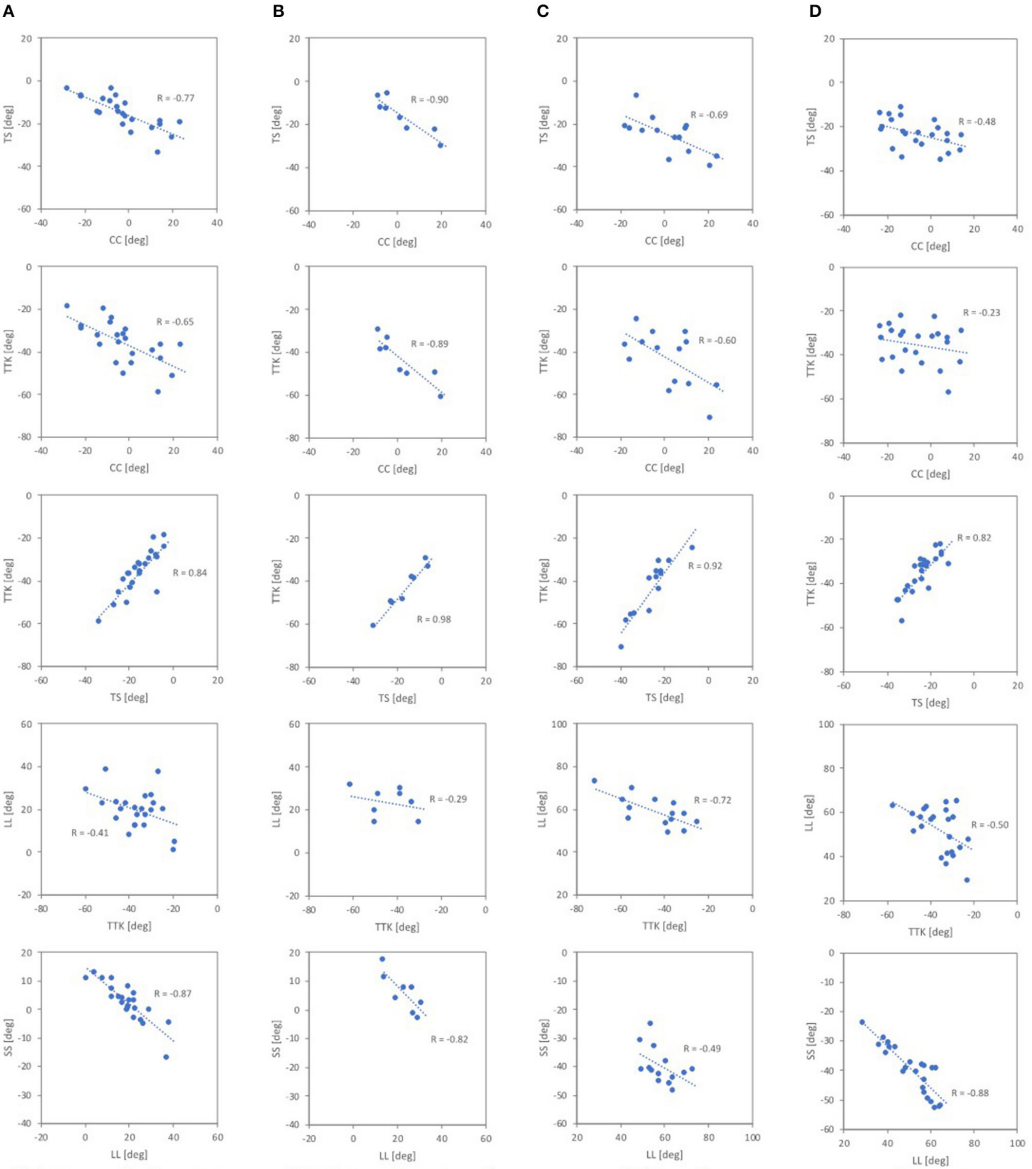
Correlations between the spinal segmental angles. **(A)** 20° seat back angle, **(B)** 25° seat back angle, **(C)** standing, **(D)** supine.

Correlations between the spinal segmental angles were looked at, obtaining the Pearson product–moment correlation coefficients. The Pearson product–moment correlation coefficient between the spinal segmental angles is summarized in Table 3 and Figure 9. For the automotive seating posture in the 20° and 25° seat back angles, correlations were observed between CC, TS, and TTK and between LL and SS. There was no correlation seen between cervicothoracic segmental angles and lumber segmental angles in this study. On the other hand, the standing posture had correlations between CC, TS and TTK, and between TTK and LL. For the supine posture, correlations were found between TS and TTK and between LL and SS.

**Table 3:** Pearson product–moment correlation coefficient R between the spinal segmental angles.

	**CC**	**TS**	**TTK**	**LL**
**(1) Automotive seating posture with the 20°seat back angle**				
TS	−0.77	–	–	–
TTK	−0.65	0.84	–	–
LL	0.11	−0.17	−0.41	–
SS	0.02	0.03	0.10	−0.87
**(2) Automotive seating posture with the 25° seat back angle**				
TS	−0.90	–	–	–
TTK	−0.89	0.98	–	–
LL	0.14	−0.21	−0.29	–
SS	0.01	0.01	−0.01	−0.82
**(3) Standing posture**				
TS	−0.70	–	–	–
TTK	−0.60	0.92	–	–
LL	−0.25	−0.49	−0.72	–
SS	0.01	0.33	−0.22	−0.49
**(4) Supine posture**				
TS	−0.48	–	–	–
TTK	−0.23	0.82	–	–
LL	−0.47	−0.19	−0.50	–
SS	0.48	0.14	0.24	−0.88

#### Automotive Seating Postures in the 20° and 25° Seat Back Angles

**Figure 10:**
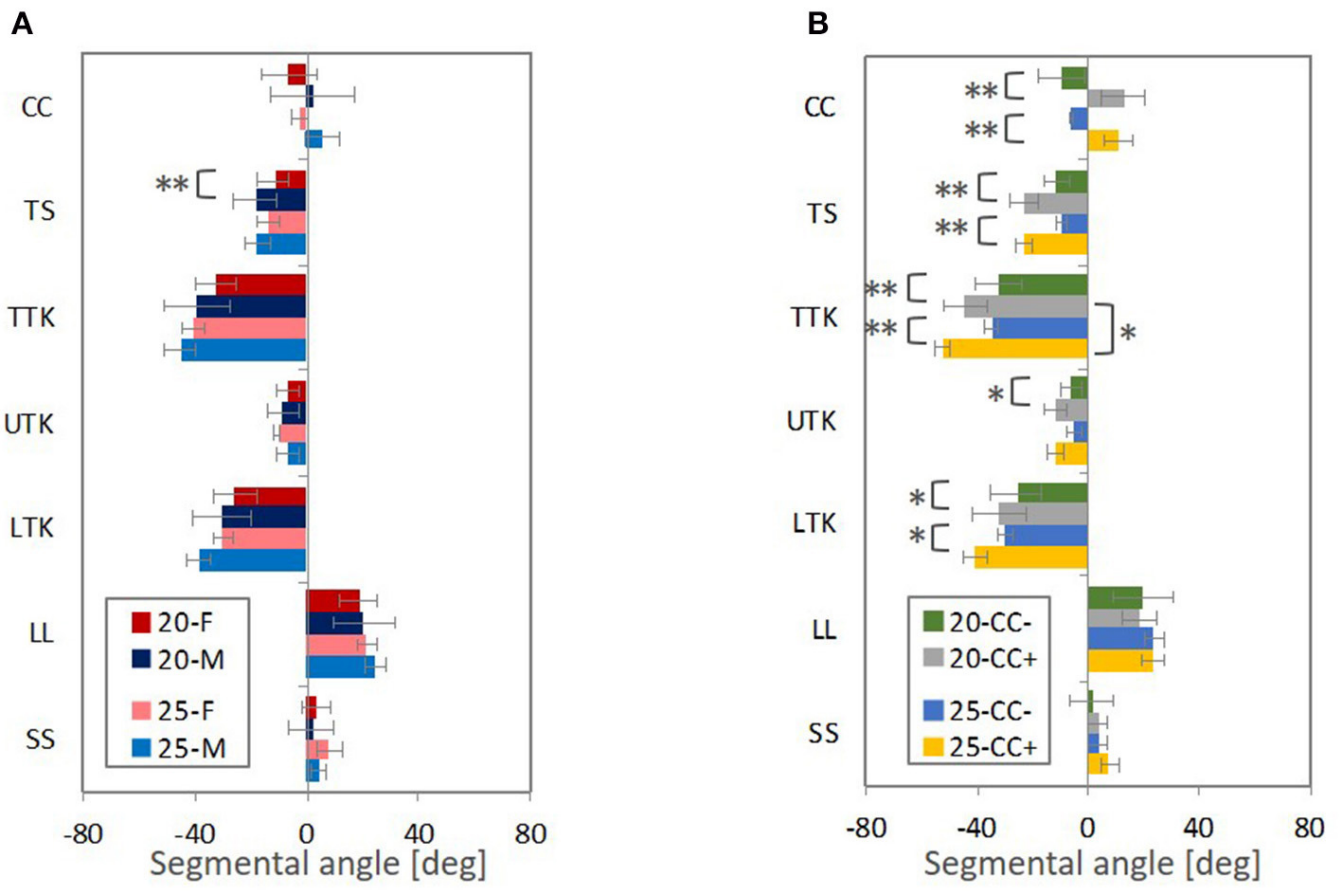
The average segmental angles with standard deviation and *p*-value from *t*-test (** < 0.05, * <0.1), **(A)** comparing male and female subjects (M and F) and **(B)** the negative CC (CC−) and positive CC (CC+). Figures in legends indicate the seat back angles from the vertical line.

The spinal segmental angles for the automotive seating postures are summarized in Figure 10. Subjects were categorized into two groups, according to the major trend of the spinal alignment patterns for the seating postures observed in the MDS analysis, based on gender and CC (cervical lordosis with positive values of CC or cervical kyphosis with negative values of CC). CC varied within the male and female groups for both seat back angles; negative CC (kyphotic) for nine females and six males and positive CC (lordotic) for two females and six males in the 20°seat back angle, and negative CC (kyphotic) for two females and two males, and positive CC (lordotic) for one female and three males in the 25° seat back angle.

In the groups of subjects based on positive and negative CCs, as shown in Figure 10B, significant differences were observed for both the 20° and 25° seat back angles in CC, TS, TTK, and LTK. The absolute average values of TS, TTK, and LTK were greater for subjects with positive CCs (lordotic) than subjects with negative CCs (kyphotic). When comparing the 20° and 25° seat back angles, the absolute average value of TTK was significantly greater for the 25° seat back angle and showed the most prominent influence of seat back inclination on spinal alignment for subjects with positive CCs (lordotic). TS and UTK indicated similar angles in both the 20°and 25° seat back angles in each group. Hence, the effect of seat back inclination may be observed most predominantly in LTK.

Likewise, for both genders, as shown in Figure 10A, the absolute values of average TTK and LTK were relatively greater for the 25° seat back angle compared with the 20° seat back angle, even though no significant difference was observed between the two seat back angles. In comparing genders, a significant difference was observed in TS for the 20° seat back angle, and the absolute values of average TS, TTK, and LTK were greater for the male subjects than the female subjects in both the 20°and 25° seat back angles. The female subjects in this study tended to negative CC (kyphotic). Findings observed between spinal alignments with positive CCs (lordotic) and negative CCs (kyphotic) may affect differences in the average gender-specific spinal alignment.

#### Standing and Supine Postures

**Figure 11:**
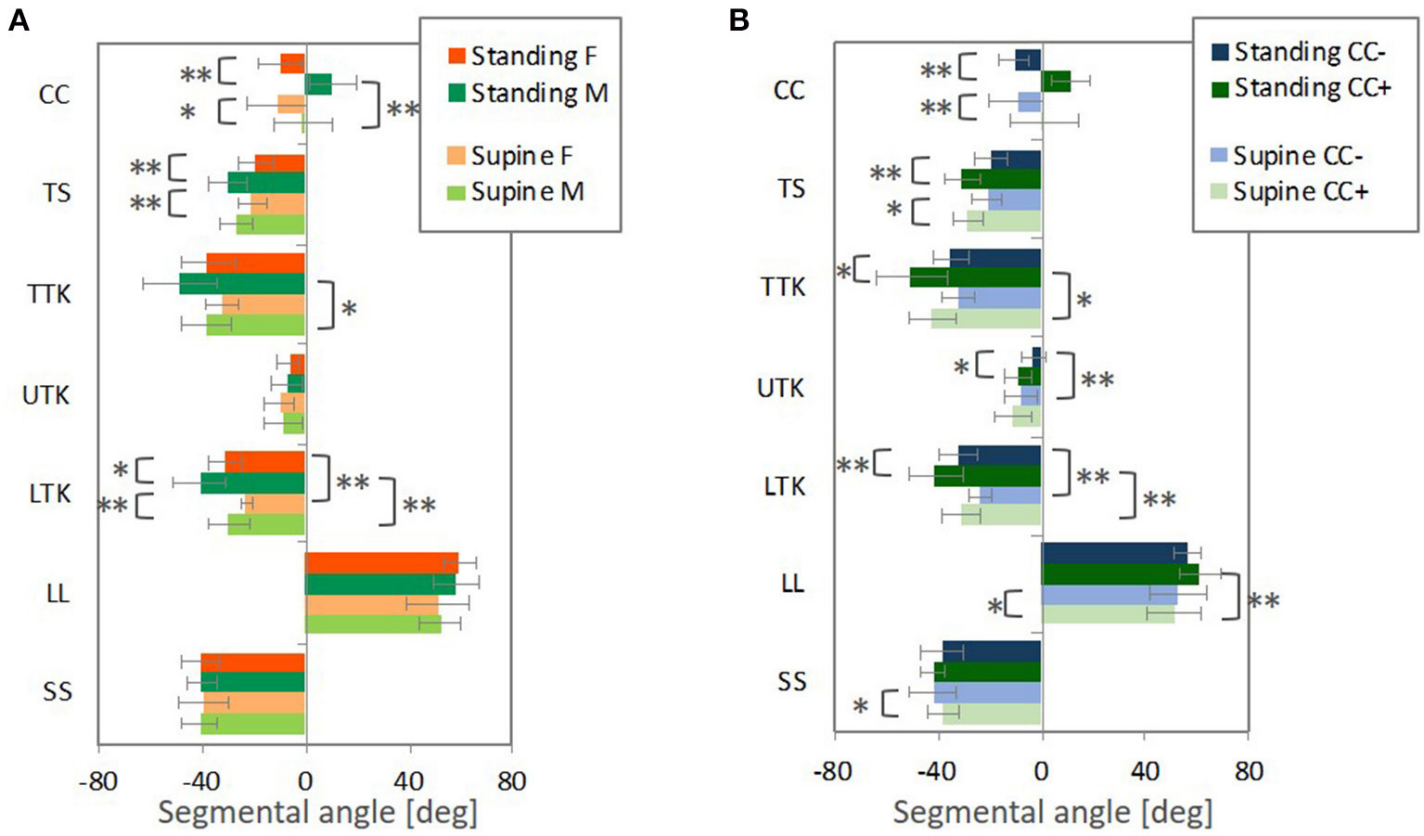
The average segmental angles with standard deviation and *p*-value from *t*-test (** < 0.05, * < 0.1), **(A)** comparing male and female subjects (M and F) and **(B)** the negative CC (CC−) and positive CC (CC+).

The spinal segmental angles for the standing and supine postures are summarized in Figure 11. Subjects were grouped based on gender and CC in a similar way to the automotive seating postures. CC varied within the male and female groups; negative CC (kyphotic) for five females and one male and positive CC (lordotic) for one female and seven males for the standing posture, and negative CC for eight females and six males, and positive CC for three females and six males for the supine posture.

In groups separating subjects into positive CC or negative CC, as shown in Figure 11B, significant differences were found in CC, TS, TTK, UTK, LTK for the standing posture, and CC, TS, LL, and SS for the supine posture. The absolute average values of TS, TTK, UTK, and LTK were greater for subjects with positive CCs (lordotic) than subjects with negative CCs (kyphotic) for both the postures. When comparing the spinal alignments in the two postures, the absolute values of average LTK were significantly greater for the standing posture than for the supine posture for subjects with both positive and negative CCs.

For both genders, as shown in Figure 11A, the absolute values of average LTK were significantly greater for the standing posture compared with the supine posture. In comparing genders, significant differences were observed in CC, TS, and LTK, and the absolute values of average TS and LTK were greater for the male subjects than the female subjects in both postures. Since the female subjects in this study tended toward negative CC (kyphotic), differences observed between spinal alignments with positive CCs and negative CCs (kyphotic) may affect the average gender-specific spinal alignment.

**Figure 12:**
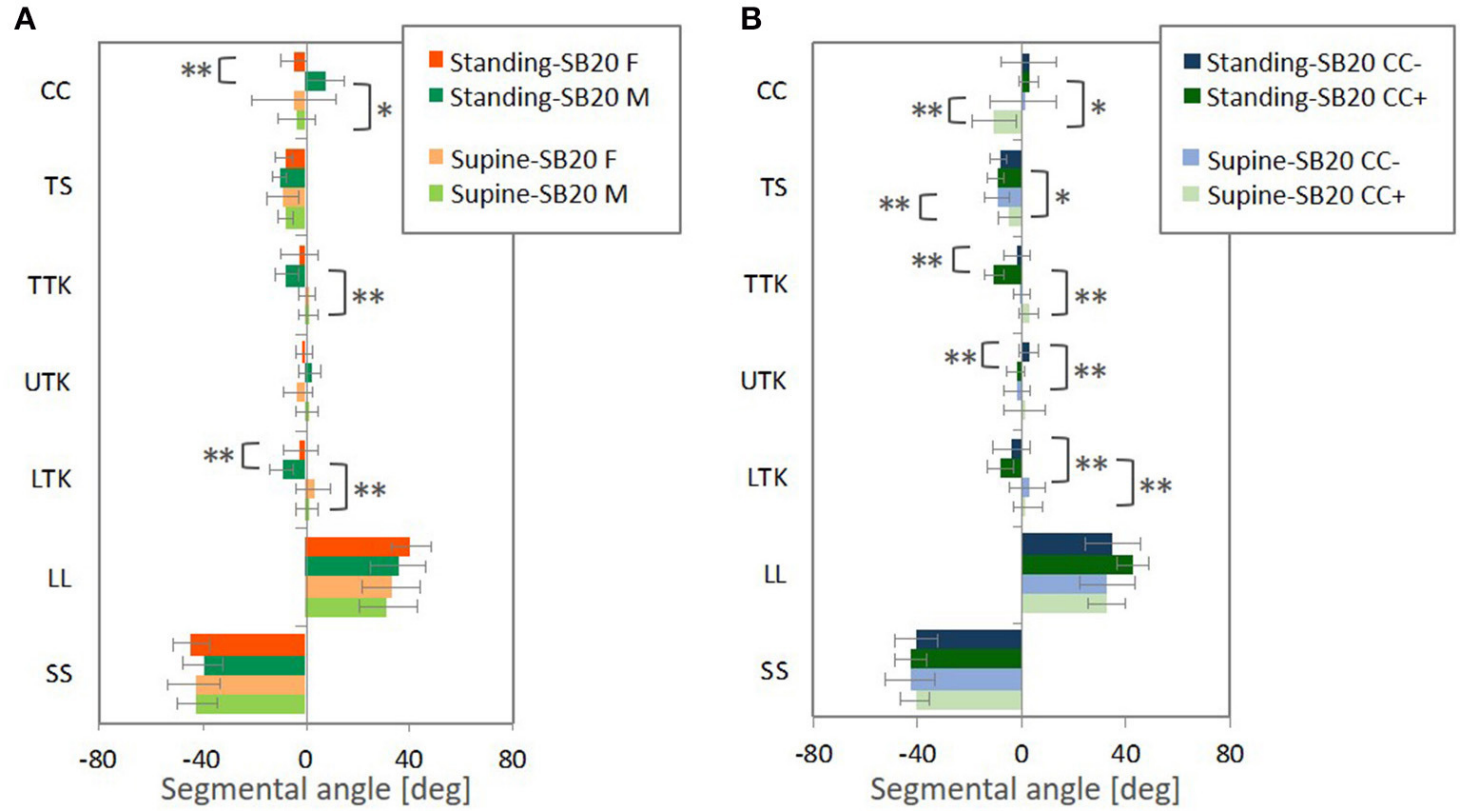
The average with standard deviation and *p*-value from *t*-test (** < 0.05, * < 0.1) for differences of the segmental angles in the standing and supine postures relative to the seating posture with the 20° seatback angle, **(A)** comparing male and female subjects (M and F) and **(B)** the negative CC (CC−) and positive CC (CC+). Differences were calculated by subtracting the segmental angles in the seating posture from the segmental angles in the standing or supine posture, individually.

Figure 12 illustrates differences in the seating posture with the seat back at 20° and the standing or supine posture. The prominent differences were found in LL and SS, followed by TS for both the postures. For the standing posture, the difference from the seating posture with the 20° seat back was significantly greater in LTK than for the supine posture, leading to more pronounced kyphosis in TTK, particularly for the male subjects and subjects with positive CCs (lordotic).

## Discussions

### Spinal Alignment in Automotive Seating Postures

The effect of seat back inclination on spinal alignment was investigated, comparing representative spinal alignment patterns in automotive seating postures with the seat back at 20° and 25° angles and standing and supine postures through MDS analyses on a data set of spinal alignment. The results of the MDS analysis indicated that the first MDS dimension, illustrating the maximum inter-subject variance, accounted for 62.5% of the total inter-subject variance of spinal alignments for the automotive seating posture with the 20° seat back angle, 72.0% for the 25° seat back angle, 61.7% for the standing posture and 53.4% for the supine posture, respectively. The first MDS dimension can explain a major part of the variety in spinal alignment for each posture. Since MDS detects meaningful underlying dimensions in a data set, spinal alignment patterns can be classified by applying an MDS analysis on a set of spinal alignment data (Mochimaru and Kouchi, 2000; Miyazaki et al., 2005). On a distribution map obtained by the MDS analysis, spinal alignments at the intersection of the 50% probability ellipse with the axes of the two MDS dimensions were estimated to interpret underlying spinal alignment patterns portrayed along each MDS dimension.

Comparing the estimated spinal alignments of the automotive seating postures along the axis of the first MDS dimension (Figures 3B, 4B), the largest variance in spinal alignment due to individual differences showed a prominent relationship between the cervical and thoracic spinal alignment. The combination varies between a slight kyphotic or almost straight cervical spine with the less-kyphotic thoracic spine to lordotic cervical spine with a more pronounced kyphotic thoracic spine.

Reed and Jones (2017) reanalysed sagittal X-ray images of the head and the cervical spine, captured in a previous study by Snyder et al. (1975), of a total of 140 female and male volunteers seated on a hard seat resembling a vehicle seat. In their principal component analysis on cervical spines, the first principal component illustrated slightly kyphotic and pronounced lordotic cervical spinal alignments obtained at ± three SDs of the principal component score, respectively, with a straighter cervical spinal alignment obtained at the mean principal component score. The results are similar to the results in this study, thus supporting the observation of cervical spinal alignments on the distribution map in this study.

The correlation analysis on the spinal segmental angles of the automotive seating postures also indicated strong negative correlations between CC and TTK, as shown in Table 3 (1) and (2) and Figures 9A,B. The MDS analyses and the correlation analyses supported a similar trend (cervical lordosis occurred with more pronounced thoracic kyphosis than cervical kyphosis). According to findings through the MDS analyses of the spinal alignment and the correlation analysis of the spinal segmental angles, spinal alignments were classified into two groups based on the CC angle in the investigation of spinal segmental angles. The absolute values of the average TS, TTK, and LTK angles were significantly greater for subjects with positive CC (lordotic) than subjects with negative CC (kyphotic) for both the 20° and 25° seat back angles, as shown in Figure 10B. In line with this, the influence of the seat back inclination on the spinal segmental angles was greater for subjects with positive CC (lordotic) than subjects with negative CC (kyphotic), indicating the most prominent influence in TTK. The comparison of the spinal alignments of the automotive seating postures estimated at the intersections of the 50% probability ellipse with the axis of the first MDS dimension on the distribution map (Figures 3B, 4B) illustrated those findings observed in the spinal segmental angles.

For the cervicothoracic region, previous studies on spinal alignment have reported that cervical lordosis tends to have a more pronounced thoracic kyphosis (Hardacker et al., 1997; Erkan et al., 2010; Ames et al., 2013; Endo et al., 2016) with greater C7 (Endo et al., 2016) and T1 inclination (Ames et al., 2013; Lee et al., 2014; Park et al., 2015) in the standing posture. Conversely, cervical kyphosis tends to have a less-kyphotic thoracic spine with smaller C7 and T1 inclination. T1 inclination has been suggested as a predictor of whole spinal alignment in the standing posture due to relationships along the cervical, thoracic and lumbar spines (Knott et al., 2010; Jun et al., 2014; Lee et al., 2015). In this study, the spinal alignment of the standing posture demonstrated consistency with these previous findings, as shown in Figures 6B, 11, and Table 3 (3). Spinal alignment trends in the automotive seating postures were similar to the previous findings in the standing posture. However, in Figure 12, differences of spinal alignment between the seating and standing postures were seen in TS and TTK including LTK, slightly in CC, due to maintaining spinal balance in the seating and standing postures, respectively.

For the lumbar region, the average LL angle indicated a similar value between subjects with positive CC (lordotic) and negative CC (kyphotic) in both the 20° and 25° seat back angle conditions, as shown in Figure 10B. The comparison of the spinal alignments estimated at the intersections of the 50% probability ellipse with the axis of the first MDS dimension on the distribution map (Figures 3B, 4B) does not illustrate a pronounced difference in the lumbar spine, such as in the cervical and thoracic spine. In addition, CC, TS, and TTK do not correlate with LL, as described in Table 3 (1) and (2).

Previous studies have reported that cervical lordosis tends to have a more pronounced thoracic kyphosis and less lumbar lordosis due to maintaining spinal balance in the standing posture (Gore et al., 1986; Roussouly and Pinheiro-Franco, 2011; Ames et al., 2013). On the other hand, another study has indicated that the cervical curvature does not have a prominent relationship with lumbar lordosis and sacral slope (Endo et al., 2016). Spinal alignment of the standing posture in this study demonstrated that CC had a negative correlation with TTK, and TTK also negatively correlated with LL. Consequently, cervical lordosis tends to have a more pronounced thoracic kyphosis and less lumbar lordosis, even though no correlation was found between CC and LL. As in the study by Endo et al. (2016), there was no correlation between LL and SS. Regarding the seating postures, the laboratory seat used in this study consisted of two stiff, flat plates. The subjects leaned in for good contact with the flat plane seat back along the entire back. Thus, the lumbar spine was straightened along with the seat back. Due to flexibility in the lumbar spine, this may not cause any significant difference in the lumbar spine between subjects with positive CC (lordotic) and negative CC (kyphotic) in both the 20° and 25° seat back angle conditions.

### Gender Differences of Spinal Alignment

Average gender-specific spinal alignments were estimated at the average gender points on the distribution map of spinal alignment. For the automotive seating postures, the average spinal alignments include an almost straight cervical and less-kyphotic thoracic spine for the female subjects, and lordotic cervical and more pronounced kyphotic thoracic spine for the male subjects, as shown in Figures 3D, 4D, 5. The average gender-specific spinal alignments in the standing posture also illustrated these trends observed in the automotive seating posture. On the distribution map of spinal alignments (Figures 3A, 4A, 6A), the average gender-specific points were almost on the axis of the first MDS dimension, located at the left side against the origin for female subjects and the right side for male subjects, within the 50% probability ellipse. The origin indicates the average of all data. Therefore, average gender-specific spinal alignments were in line with the trend observed along the first MDS dimension, with a smaller difference than that between the estimated spinal alignments at the intersections of the 50% probability ellipse and the axis of the first MDS dimension.

In the investigation of spinal segmental angles, the average CC angle was greater for the male subjects than for the female subjects in the four postures, as shown in Figures 10A, 11A. Also, the absolute values of the average TS, TTK, and LTK angles were greater for the male subjects than for the female subjects. The comparison of the estimated average gender spinal alignments (Figures 3D, 4D, 6D, 7D) illustrated similar findings in the spinal segmental angles. However, for the automotive seating postures, only the TS in the 20° seat back angle condition indicated a significant difference between genders, whereas significant differences were observed in CC, TS, and LTK for the standing and supine postures.

As reported in previous studies on the variation in cervical spinal alignment in the standing or upright seating postures (Helliwel et al., 1994; Hardacker et al., 1997; Matsumoto et al., 1998), gender is an independent factor that correlates significantly with non-lordosis. Women are more likely to present non-lordosis (kyphotic or straight). Conversely, men present more pronounced lordosis. In this study, the average gender-specific spinal alignments in the cervical spine were almost straight for the female subjects and lordotic for the male subjects. The average CC angle was positive for the male subjects, whereas negative for the female subjects. Findings in this study correlate with previous studies.

At the cervicothoracic junction, relationships along the cervical spinal alignment, C7 or T1 inclination, and thoracic kyphosis have been investigated with focussing on gender differences (Lee et al., 2014; Park et al., 2015; Endo et al., 2016). A decrease in the C7 and T1 inclination is associated with kyphosis, or an increase in hypo-lordosis in cervical spinal alignment and less kyphosis in thoracic spinal alignment. Men tend to have greater C7 and T1 inclination (more forward-inclined C7 and T1), while women tend to have shorter C7 and T1 inclination (less forward-inclined C7 and T1). With decreasing T1 inclination, women are more likely to present a hypo-lordotic or kyphotic cervical spine and a less kyphotic thoracic spine than men. The average female spinal alignments in this study portrayed a less forward inclination around C7 and T1 displaying a straighter cervical and thoracic spine than the average male spinal alignment. The average CC angle and the absolute angles of the average TS, TTK, and LTK were smaller for female subjects than for male subjects. The gender differences observed in this study are in agreement with the above-mentioned previous studies.

Since CC of the female subjects tended to be negative, the trends observed in a comparison between subjects with negative (kyphotic) and positive (lordotic) CC (Figures 10B, 11B) might affect differences between genders (Figures 10A, 11A). Therefore, the average female exhibited less TS with less-kyphotic thoracic alignment and thus straighter cervicothoracic spinal alignment than the average male, despite no significant differences observed in CC, TTK, and LTK for the automotive seating postures. In addition, the influence of the seat back inclination on the spinal segmental angles was greater for subjects with positive CC (lordotic) than subjects with negative CC (kyphotic), indicating the most prominent influence in TTK, including LTK. This finding might have an impact on differences in the influence of the seat back inclination between genders. Indeed, the differences in LTK between the seating posture with the 20° seat back angle and the standing or supine posture were significantly greater for the male subjects. In the MDS analyses, the average gender-specific points were almost on the axis of the first MDS dimension on the distribution map of spinal alignment for the two seating and standing postures. Consequently, the average female point was positioned opposite the average male point across the origin, which may suggest gender as one of the factors affecting the largest inter-individual variance in spinal alignment.

The study of the lumbar region only revealed minor average gender-specific spinal alignment and LL differences. In a report by Endo et al. (2014), lumbar lordosis is significantly greater for women than men in the upright seating position, while the present study focused on an automotive seating posture instead of an upright seating posture. As mentioned in the preceding Section, subjects in this study were seated deeply on a stiff laboratory seat leaning the entire back against the flat plane seat back. This caused the lumbar spine to straighten and the seat back, showing no significant gender differences, such as in the upright seating posture.

## Limitations

A limited number of subjects, in their 20s and 30s, were selected based on the average Japanese body sizes (Ministry of Education, 2013), the mid-sized female and male (Schneider et al., 1983). All subjects were close to the average body size in their gender. Due to the seat consisting of two flat plates, the spinal alignments observed in this study will likely not be affected by the seated height, although age and BMI might affect spinal alignment. A larger number of subjects will be needed to generalize spinal alignment patterns in other specific ages and body sizes.

The laboratory seat used in this study was designed to exclude the influence of seat properties (foam, frame stiffness and its distribution, etc.) and the external shape of the seat back and seat pan. This design was neutral to body size differences between individuals and genders compared with a regular car seat. However, these seat specifications may influence spinal alignment. Variations in designs of commercially available car seats would need to be considered for future studies in more realistic situations.

This study recruited only asymptomatic volunteers. Future studies could also compare the findings obtained in this study with whole spinal alignments of patients suffering from spinal pathologies. Providing the differences of whole spinal alignment between asymptomatic volunteers and patients to a human body FE model, computational simulations may show different vertebral kinematics and provide better knowledge of spinal injury mechanisms.

In addition, MRI scans take more test duration time than CT and X-rays scans. The test duration time may affect postural stability. Likewise, the duration of driving and the type of route driven may affect postural changes (Ghaffari et al., 2018), which is a topic of future study on spinal alignment of automotive seating postures.

## Conclusions

The spinal alignment in the 25° seat back angle displayed more pronounced thoracic kyphosis than in the 20° seat back. The most prominent influence of seat back inclination on segmental angles appeared in TTK, including LTK when categorizing spinal alignments into two groups based on CC. The differences of TTK and LTK between the two seat back angles and between the seating posture with the 20° seat back and the standing posture were greater for spinal alignments with positive CCs than for spinal alignments with negative CCs. In this study, the female subjects tended negative CC. Some of the differences between average gender-specific spinal alignments may be explained by the findings observed in the differences between positive CC and negative CC spinal alignments.

## Data Availability Statement

The original contributions presented in the study are included in the article/supplementary material, further inquiries can be directed to the corresponding author/s.

## Ethics Statement

The studies involving human participants were reviewed and approved by Ethical Committees of Shiga University of Medical Science in Japan, Hospital Universitario HM Montepríncipe (Fundación de Investigación HM Hospitales) in Spain, Japan Automobile Research Institute and Tokyo Institute of Technology in Japan. The patients/participants provided their written informed consent to participate in this study.

## Author Contributions

FS outlined this study. FS, SM, AF, MS, and SS conducted the MRI scans. FS and YM jointly analyzed and presented all data included in the paper. The paper was written by FS and reviewed by all authors. All authors contributed to the article and approved the submitted version.

## Conflict of Interest

The authors declare that the research was conducted in the absence of any commercial or financial relationships that could be construed as a potential conflict of interest.

## Publisher's Note

All claims expressed in this article are solely those of the authors and do not necessarily represent those of their affiliated organizations, or those of the publisher, the editors and the reviewers. Any product that may be evaluated in this article, or claim that may be made by its manufacturer, is not guaranteed or endorsed by the publisher.
